# An Experimental Research into Surgical Shock

**Published:** 1899-12

**Authors:** 


**An Experimental Research into Surgical Shock.** By GEORGE W. CRILE, M.D. Pp. 160. Philadelphia: J. B. Lippincot Company. [1899.]

A brief enumeration of the theories on the subject of surgical shock forms the introduction to this essay, which was awarded the Cartwright Prize for 1897. Then, after a description of the apparatus used, over one hundred and thirty experiments, carried out for the most part on dogs, are detailed. In these experiments cannulas were fixed into various arteries, and the blood pressure estimated after injuries of every conceivable description were inflicted on almost every tissue and organ of the body. Following on the record of the experiments is a summary of the results of these injuries on the various parts affected. Some well-known facts as to the causes, prevention, and treatment of shock are then narrated. The final conclusion arrived at is that surgical shock is mainly due to impairment or break-down of the vaso-motor mechanism. The book represents a vast amount of labour, which is chronicled in a very elaborate and graphic manner by means of a large number of diagrams and charts.

